# Two assumptions of the prior event rate ratio approach for controlling confounding can be evaluated by self-controlled case series and dynamic random intercept modeling

**DOI:** 10.1016/j.jclinepi.2024.111511

**Published:** 2024-11

**Authors:** Yin Bun Cheung, Xiangmei Ma, Grant Mackenzie

**Affiliations:** a Programme in Health Services & Systems Research, Duke-NUS Medical School, 8 College Road, Outram Park, Singapore 169857; b Centre for Quantitative Medicine, Duke-NUS Medical School, 8 College Road, Outram Park, Singapore 169857; c Tampere Center for Child, Adolescent and Maternal Health Research, Tampere University, Arvo Ylpön katu 34, Tampere 33520, Finland; d Medical Research Council Unit The Gambia at London School of Hygiene & Tropical Medicine, Fajara, P.O. Box 273, The Gambia; e New Vaccines Group, Murdoch Children's Research Institute, Flemington Road, Melbourne, Victoria 3052, Australia; f Department of Paediatrics, University of Melbourne, Parkville, Victoria 3010, Australia

**Keywords:** Confounding, Dynamic random intercept model, Observational studies, Prior event rate ratio, Self-controlled case series, Real-world evidence

## Abstract

**Objectives:**

The prior event rate ratio (PERR) is a recently developed approach for controlling confounding by measured and unmeasured covariates in real-world evidence research and observational studies. Despite its rising popularity in studies of safety and effectiveness of biopharmaceutical products, there is no guidance on how to empirically evaluate its model assumptions. We propose two methods to evaluate two of the assumptions required by the PERR, specifically, the assumptions that occurrence of outcome events does not alter the likelihood of receiving treatment, and that earlier event rate does not affect later event rate.

**Study Design and Setting:**

We propose using self-controlled case series (SCCS) and dynamic random intercept modeling (DRIM), respectively, to evaluate the two aforementioned assumptions. A nonmathematical introduction of the methods and their application to evaluate the assumptions are provided. We illustrate the evaluation with secondary analysis of deidentified data on pneumococcal vaccination and clinical pneumonia in The Gambia, West Africa.

**Results:**

SCCS analysis of data on 12,901 vaccinated Gambian infants did not reject the assumption of clinical pneumonia episodes had no influence on the likelihood of pneumococcal vaccination. DRIM analysis of 14,325 infants with a total of 1719 episodes of clinical pneumonia did not reject the assumption of earlier episodes of clinical pneumonia had no influence on later incidence of the disease.

**Conclusion:**

The SCCS and DRIM methods can facilitate appropriate use of the PERR approach to control confounding.

**Plain Language Summary**

The prior event rate ratio is a promising approach for analysis of real-world data and observational studies. We propose two statistical methods to evaluate the validity of two assumptions it is based on. They can facilitate appropriate use of the prior even rate ratio.

**What is new?**

**Key findings**
• The self-control case series and dynamic random intercept modeling methods can be used to evaluate two assumptions of the prior event rate ratio method, specifically, the assumption that occurrence of outcome events does not alter the likelihood of receiving treatment, and the assumption that earlier event rate does not affect later event rate.

**What this adds to what was known?**
• The prior event rate ratio method has a potential to control confounding in observational studies and real-world evidence research, but its validity relies on a series of assumptions and there has been no guidance on how to empirically evaluate them.

**What is the implication and what should change now?**
• The proposed methods should be used to evaluate the assumptions prior to application of the prior event rate ratio method.

## 1 Introduction

The prior event rate ratio (PERR) approach has been proposed to control measured or unmeasured confounders in analysis of real-world data and observational studies in which the outcomes are nonterminal events [1,2]. Tannen et al applied the PERR to estimate the efficacy of several drugs on the incidence of cardiovascular events using electronic health records [1]. They designed the studies such that the treatments were comparable to randomized controlled trials of the same or similar treatments. They compared the PERR with multivariable regression and propensity score methods for controlling confounding. Using previously published efficacy estimates from randomized controlled trials as the gold standard, they found that PERR out-performed the other methods in terms of producing efficacy estimates that were close to the gold standard estimates. Two other observational studies similarly found PERR superior to alternative methods for controlling confounding [3,4]. This approach has also been applied to study the safety and efficacy of a variety of other biopharmaceutical products [[5], [6], [7], [8], [9], [10], [11]].

The core idea of the PERR is to partition person-time of persons who have been treated with the product under investigation during an observation period into two sections: prior to and after initiation of treatment. Each treated person is matched to K (K ≥ 1) control persons who never received the treatment. The treated person's date of treatment initiation is the index date for partitioning the matched control's person-time into prior and postperiods. The matching procedure is to ensure that the untreated controls have index dates that are commensurate with the treatment dates of the treated persons. The hazard of the outcome event is then compared between the two groups of people separately in the prior period and in the postperiod, generating two hazard ratios (HRs), ${\text{HR}}_{\text{prior}}$ and ${\text{HR}}_{\text{post}}$, respectively. The PERR aims to cancel out confounding by estimating treatment effect as the ratio of the two HRs:

$${\text{HR}}_{\text{PERR}}={\text{HR}}_{\text{post}}/{\text{HR}}_{\text{prior}}$$

Despite promising findings from previous studies and rising popularity, validity of the PERR depends on a series of assumptions [1,5,12,13]. Previous studies have relied on prior knowledge to determine the validity of the assumptions. There is a lack of strategy to empirically evaluate the validity of these assumptions.

This article focuses on two of the assumptions about the relationship of events over time: (1) occurrence of the outcome event does not alter the probability of receiving treatment and (2) outcome event rate in the prior period does not affect outcome event rate in the postperiod. We propose that the self-controlled case series (SCCS) method and dynamic random intercept modeling (DRIM) can be used to assess the plausibility of these two assumptions, respectively. We will use data on pneumococcal vaccination and clinical pneumonia in Gambian children to illustrate.

## 2 Materials and methods

### 2.1 Self-controlled case series

The SCCS is commonly used in the monitoring of drug and vaccine safety. Whitaker et al gave an excellent introduction to the method [14]. Further details and R packages can be found in Farrington et al [15] Briefly, the SCCS analyzes data from cases only, that is, people who have experienced the outcome event. It uses conditional Poisson regression to compare the incidence of the outcome event between time intervals defined in relation to the timing of treatment, such as the first 28 days postvaccination being a risk window vs. time before and over 28 days after vaccination as a reference period. Exponentiation of the regression coefficients gives relative incidence in the risk window as compared to the reference period. Since the conditional Poisson regression makes comparison between time intervals within persons, it automatically prevents confounding by all time-constant covariates such as ethnicity. The method requires appropriate model specification to adjust for time-varying covariates such as age [14]. In the presence of time-varying covariates, cases that never received the treatment can also be included into the analysis, which improves the precision of the estimates [16]. If a case had multiple episodes of the outcome event during the study period, the typical recommendation is to include only the first episode in the analysis. Otherwise an additional assumption of absence of event dependence is required [14,15]. The method was originally developed for rare events, but unbiased estimation of the relative incidence of nonrare events can be achieved by adjustment for age or time intervals as time-varying covariates [17,18].

In SCCS studies of vaccine safety, a person may be treated multiple times, that is, multiple doses of the vaccine. The outcome is an episode of an adverse event. To evaluate the PERR method's assumption that occurrence of prior events does not affect the probability of treatment, we reverse the role of “treatment” and “outcome”. For example, in the study of pneumococcal vaccination in Gambian infants that we will discuss shortly, we defined pneumococcal vaccination as the outcome and clinical pneumonia episodes as the treatment. As with typical SCCS analysis, the treatment (now clinical pneumonia episodes) may occur multiple times, while only the first episode of the outcome event (first dose of pneumococcal vaccine) is included.

### 2.2 Dynamic random intercept model

An excellent review of dynamic random intercept modelling (DRIM) was given by Skrondal and Rabe-Hesketh [19]. Examples of DRIM in medical research include evaluation of whether wheezing affected the chance of future wheezing [19], and persistence of a child's adherence to an immunization schedule [20]. An important issue to note is that standard random intercept models with a lagged response (ie, outcome in the previous time interval) as a time-varying independent variable can suffer serious bias due to the “initial conditions problem”. Skrondal and Rabe-Hesketh called this a naïve model [19]. The initial conditions problem arises when the first response observed during a study period is affected by the random intercept and unobserved responses prior to the start of the study. The naïve model tends to bias toward underestimating the variance of the random intercept and overestimating the coefficient for the lagged response, generating a false impression that the prior events affect later event rate. An uncommon situation in which the initial conditions problem is absent occurs when the study period starts from birth such that there are no unobserved prior responses. In this case the degree of bias for the naïve model is affected by the proportion of positive response among the first observations and the degree of association between the first and later responses [21,22].

Unbiased methods for studying lagged response in the presence of the initial conditions problem are well-established for continuous and binary outcomes. To evaluate whether prior event rate affects later event rate, we propose to partition follow-up time into equal length intervals and define the outcome (clinical pneumonia in our example) in each interval as either no event ($y_{i,j}=0$) or at least one event ($y_{i,j}=1$), where subscripts *i* and *j* index the *i*-th person and *j*-th time interval (j=0,1,2,…), respectively. A lagged response that represents past occurrence of the outcome is $lag\left(y_{i,j}\right)=y_{i,j-1}$, which is not defined for the first interval (j=0). We propose to apply the first-order autoregressive (AR1) dynamic random intercept model of Aitkin and Alfo [23], which was recommended by the review of Skrondal and Rabe-Hesketh [19]. Briefly, it is a generalized linear latent and mixed model that jointly models the first $\left(y_{i,0}\right)$ and subsequent responses $\left(y_{i,j};j\geq 1\right)$. It requires a minimum of three intervals. It can be estimated using, for example, Stata's *gllamm* or *gsem* commands. Exponentiation of the regression coefficient for the lagged response gives the odds ratio for the magnitude of association between outcomes in the previous and present time intervals. Details of the DRIM model and Stata *gsem* codes for the example study are given in Appendix 1.

### 2.3 Pneumococcal vaccination and clinical pneumonia in Gambian children

Pneumococcal conjugate vaccine (PCV) is efficacious in reducing the incidence of invasive pneumococcal disease and radiological pneumonia with consolidation [24]. It has a smaller level of efficacy in reducing the incidence of clinical pneumonia defined according to clinical signs and age-specific respiratory rate cut-offs [25,26]. In low-income settings where laboratory and x-ray facilities are limited, data on clinical pneumonia are likely more available for real-world studies. For illustration purposes, we will analyze data of infants up to the age of 12 months from a population-based demographic and disease surveillance system in Upper River Region, The Gambia, West Africa. The country started to deliver 13-valent PCV in May 2011. This illustration included infants born between June 2011 and December 2013. Details of the surveillance system have been previously published [26,27].

While infants with severe illness are usually discouraged from vaccination until they recover, they may receive PCV if they only have mild illness according to clinical judgment [28,29]. Therefore it is uncertain whether clinical pneumonia might have a short-term impact in reducing the chance of receiving PCV. On the other hand, parents of children who have suffered pneumonia may have stronger motivation to have their children vaccinated after recovery. We illustrated the use of SCCS to evaluate whether clinical pneumonia affected the likelihood of receiving PCV. Clinical pneumonia was as defined in previous publications [25,26]. Receipt of the first dose of PCV (PCV1) was the outcome in the analysis. We defined two risk windows: 1-14 days and 15-42 days after the date of diagnosis of clinical pneumonia. Times before and at least 42 days after an episode of clinical pneumonia formed the reference period. The first 14-day window was chosen to assess whether there was a short-term reduction of the chance of vaccination. The next 28-day window was chosen to assess whether clinical pneumonia would promote PCV uptake after recovery, assuming that such motivation would not last longer. If a child had two episodes of clinical pneumonia within a period less than 42 days, the overlapping duration of the 15–42 day interval after the earlier episode and 1–14 day interval after the later episode was counted only as under the influence of the later episode. Age was adjusted for as a time-varying covariate, in 2-month intervals.

Episodes of infectious diseases may induce acquired immunity and therefore reduce subsequent disease incidence. But clinical pneumonia has a diverse etiology and this relationship is uncertain. We illustrated the use of DRIM (and naïve model) to evaluate whether occurrence of clinical pneumonia in one time interval might affect the chance of clinical pneumonia in the next interval. We partitioned the observation time from birth through infancy into six 60-day intervals, each with a binary response of whether there was no episode $\left(y_{i,j}=0\right)$ or at least one episode $\left(y_{i,j}=1\right)$ of clinical pneumonia. The lagged response was $lag\left(y_{i,j}\right)=y_{i,j-1}$. Gender and mother's education were time-constant covariates. Age (in months) and distance from residence to nearest health center (km) were time-varying covariates as per their values at the beginning of intervals.

To illustrate the impact of the problem in more general situations that the first true response may be unobserved and caution against the use of the naïve model, we also analyzed the data after exclusion of the first 60-day interval.

To illustrate PERR estimation, we used having received at least one dose of PCV as the exposure. One-to-one match by gender, mother's education and distance to nearest health center (tertiles) was used to generate pairs of exposed and unexposed infants and index dates for the latter. In matching for mother's education, we combined Basic and Secondary as one category and Madrassa/Quranic and Other as another. We used age as the time-scale in the Cox models for estimation of ${\text{HR}}_{p\text{rior}}$ and ${\text{HR}}_{\text{post}}$, and bootstrapping with 1000 replicates to generate confidence interval for ${\text{HR}}_{\text{PERR}}$.

To avoid repeated counting of a disease episode, it is common that infectious disease research requires a certain number of days since diagnosis of the previous disease episode to pass before a new episode can be counted. Previous studies of pneumonia defined this period as 30 days [25,26], which we followed. The binary responses $y_{i,j}$ indicated the outcome in a 60-day interval. If an episode of clinical pneumonia occurred within the first 30 days of the 60-day interval, the 30-day gap would not affect the data structure. Otherwise, there must be some duration of time free of new episodes after this 60-day interval. In this case, we define the next 60-day interval as starting from the end of the 30-day gap. We did not allow the next 60-day interval to include the unfinished part of the 30-day gap because that would imply a smaller odds of the outcome in the next interval due to less time to observe it, which would induce a negative association between $y_{i,j-1}$ and $y_{i,j}$.

## 3 Results

There were 15,304 infants born during the study period. Table 1 shows the descriptive characteristics of the infants and subsets of them to be included in the SCCS, DRIM, and PERR analyses.

**Table 1:** Descriptive statistics of Gambian infants, Upper River Division, June 2011 to December 2013

Variables	All (*N* = 15,304)	SCCS subset (*N* = 12,901)	DRIM subset (*N* = 14,325)	PERR subset (*N* = 4038)
Gender				
Male	7763 (50.7%)	6541 (50.7%)	7258 (50.7%)	2020 (50.0%)
Mother's education				
None	2271 (14.8%)	1873 (14.5%)	2141 (15.0%)	686 (17.0%)
Basic	1208 (7.9%)	1049 (8.1%)	1143 (8.0%)	259 (6.4%)
Secondary	365 (2.4%)	328 (2.5%)	333 (2.3%)	59 (1.5%)
College/university	1701 (11.1%)	1489 (11.5%)	1635 (11.4%)	348 (8.6%)
Madrassa/Quranic	8912 (58.2%)	7708 (59.8%)	8433 (58.9%)	2266 (56.1%)
Other	847 (5.5%)	454 (3.5%)	640 (4.5%)	420 (10.4%)
Distance^a^				
Mean (SD)	6.08 (5.99)	6.10 (6.05)	6.12 (6.00)	6.23 (5.94)

PERR, prior event rate ratio.

a Distance to nearest health center (km) is a time-varying covariate due to movement; value at baseline is shown.

### 3.1 Self-controlled case series

Among the 15,304 infants, 12,902 received PCV1. Distribution of the age at PCV1 was positively skewed, with median 2.7 months, and 1st and 99th percentiles 1.2 and 9.0 months, respectively. One of the 12,902 vaccinated infants had no variation in exposure (ie, no record of clinical pneumonia and entered and exited the surveillance system within the same age interval). This observation was not informative in the SCCS analysis and was excluded. Among the 12,901, 1385 had at least one episode of clinical pneumonia during infancy. SCCS analysis adjusted for age effect estimated the relative incidence of receiving PCV1 as 1.14 (95% CI = 0.89 to 1.46; *P* = .313) and 0.88 (0.69 to 1.12; *P* = .301) during the 1-14 days and 15-42 days risk windows, respectively. There was no evidence of clinical pneumonia affecting PCV uptake.

### 3.2 Dynamic random intercept modeling

Among the 15,304 infants, 14,325 had complete observations (six 60-day observations). Among them, 1,275, 167 and 34 had 1, 2, and ≥3 episodes of clinical pneumonia. Table 2 shows the results of DRIM and naïve model analysis. Using longitudinal data from birth, having adjusted for covariates, clinical pneumonia in the previous interval had little association with that in the present interval, with odds ratios exp (0.08) = 1.08 (0.81 to 1.42; *P* = .599) and exp (0.10) = 1.11 (0.84 to 1.48; *P* = .477) estimated from the DRIM and naïve model, respectively. There was no evidence of association of clinical pneumonia over time.

**Table 2:** Illustration of naive modelling and dynamic random intercept modelling (DRIM) with logit link function, using complete data from birth (six 60-day intervals) and excluding the first interval (to generate initial conditions problem); clinical pneumonia in Gambian infants

Parameters	Complete data		Exclude first interval	
	DRIM coef. (95% CI)	Naïve coef. (95% CI)	DRIM coef. (95% CI)	Naïve coef. (95% CI)
Lagged response	0.08 (−0.21, 0.36)	0.10 (−0.18, 0.39)	0.08 (−0.22, 0.38)	0.58 (0.20, 0.97)
Gender				
Male	0.32 (0.21, 0.44)	0.32 (0.21, 0.44)	0.28 (0.16, 0.41)	0.27 (0.15, 0.39)
Mother's education				
None	0	0	0	0
Basic	0.20 (−0.04, 0.44)	0.20 (−0.04, 0.44)	0.25 (−0.01, 0.51)	0.24 (0.02, 0.49)
Secondary	0.06 (−0.32, 0.45)	0.07 (−0.32, 0.46)	−0.01 (−0.45, 0.43)	−0.01 (−0.44, 0.42)
College/university	−0.25 (−0.49, −0.01)	−0.25 (−0.49, −0.01)	−0.24 (−0.50, 0.02)	−0.23 (−0.48, 0.02)
Madrassa/Quranic	0.07 (−0.10, 0.24)	0.07 (−0.10, 0.24)	0.04 (−0.15, 0.23)	0.03 (−0.15, 0.21)
Other	−0.22 (−0.54, 0.11)	−0.21 (−0.54, 0.11)	−0.32 (−0.71, 0.07)	−0.32 (−0.69, 0.06)
Age^a^				
(months)	−0.04 (−0.05, −0.02)	−0.04 (−0.05, −0.02)	−0.03 (−0.05, 0.00)	−0.03 (−0.05, 0.00)
Distance^a^				
(km)	−0.07 (−0.08, −0.06)	−0.07 (−0.08,-0.06)	−0.07 (−0.08, −0.06)	−0.06 (−0.08, −0.05)
Intercept	−4.32 (−4.61, −4.03)	−4.32 (−4.61, −4.02)	−4.27 (−4.62, −3.91)	−4.11 (−4.47, −3.74)
Variance^b^	1.33 (1.06, 1.67)	1.31 (1.04, 1.66)	1.30 (0.96, 1.76)	0.88 (0.54, 1.44)

a Age and distance to nearest health center are time-varying covariate with values defined as at the start of each time interval.

b Variance of random intercepts.

There were only 32 cases (0.22%) in the first interval as opposed to about 300 cases in each of the subsequent intervals. The tetrachoric correlation between each response and its immediate next response, $corr\left(y_{i,j},y_{i,j+1}\right)$, were similar across all *j* (*j* = 0,1,2,3,4), in the range of 0.22 to 0.28 (Table 3). However, the tetrachoric correlation between the first and later responses, $corr\left(y_{i,0},y_{i,j+k}\right),k\geq 2$, was much weaker than that among the other responses. This indicates that the determinants of clinical pneumonia in the earliest period were likely different from the later periods. In this data pattern, the bias in the naïve model is limited [21,22].

**Table 3:** Tetrachoric correlation between responses in the six 60-day intervals

Interval	$y_{i,0}$	$y_{i,1}$	$y_{i,2}$	$y_{i,3}$	$y_{i,4}$	$y_{i,5}$
$y_{i,0}$	1.00					
$y_{i,1}$	0.26	1.00				
$y_{i,2}$	0.14	0.28	1.00			
$y_{i,3}$	0.15	0.24	0.26	1.00		
$y_{i,4}$	0.23	0.26	0.27	0.28	1.00	
$y_{i,5}$	0.17	0.22	0.24	0.20	0.22	1.00

To highlight the pitfall of naïve analysis in the presence of the initial conditions problem, we also analyzed the data after exclusion of the first 60-day interval, to mimic situations in which there are unobserved responses prior to study onset. The DRIM gave results similar to the analysis of the complete data. The naïve model showed the expected bias of overestimation of the odds ratio, exp (0.58) = 1.79 (1.22–2.63; *P* = .003), and underestimation of the variance of the random intercepts, leading to a false conclusion that earlier events increased later event rate.

### 3.3 Prior event rate ratio

Among the 15,304 infants, 350 had less than 2 months’ observation time in the surveillance system due to out-migration or death. They were excluded from PERR analysis. Among those eligible for the analysis, 12,935 did and 2019 did not receive PCV. The matching procedure described in Section 2.3 formed 2019 pairs of infants. Median age at vaccination and index age for partitioning prior- and postperiods were 2.7 months. Table 4 shows the number of clinical pneumonia and person-years by groups and periods. ${\text{HR}}_{\text{post}}$ was 1.01 (0.83–1.24), indicating no difference between vaccination groups in the postperiod. However, ${\text{HR}}_{p\text{rior}}$ was 2.20 (1.30–3.71), showing that infants who would later be vaccinated were at higher risk of pneumonia to begin with. The PERR estimate were 1.01/2.20 = 0.46 (0.25–0.79; *P* = .006), indicating PCV effectiveness.

**Table 4:** Numbers of clinical pneumonia episodes (numerator) and person-years (denominator) by groups and periods, and hazard ratios by periods

Period	Vaccinated	Unvaccinated	HR (95% CI)
Prior	46/506	20/504	2.20 (1.30 to 3.71)
Post	220/1508	184/1357	1.01 (0.83 to 1.24)

HR, hazard ratio; CI, confidence interval.

## 4 Discussion

The PERR is a promising approach to control confounding. Nonetheless, its validity relies on a series of assumptions, two of which have been discussed here. We propose using the SCCS and DRIM to evaluate these two assumptions.

The strength of our proposal is that the SCCS and DRIM are well-established methods which can be implemented using existing software [14,15,19].

The main limitation of our proposal is that the SCCS and DRIM require specifications according to the research context and themselves involve assumptions. For example, SCCS requires specification of the number and width of risk windows after the exposure. In the case study of clinical pneumonia (exposure) and PCV (outcome) we set risk windows up to 42 days after exposure, assuming that the impact of the exposure would not last long, if any. Careful consideration of such specifications according to the research context is needed. The DRIM in the current literature only allows for a first-order lagged response, assuming that $y_{i,j-k}\;\left(k\geq 2\right)$ have no association with $y_{i,j}$. Methodological research to generalize the method to allow for k≥2 is needed.

Furthermore, to be precise, the second PERR assumption that we considered is that event rate in the prior period does not affect event rate in the postperiod in both the treatment and control groups. The DRIM does not directly test this assumption. Instead, it evaluates the association of outcomes across at least three time intervals; the model is not estimable if there are only two intervals [19]. Hypothetically, we may imagine a situation in which there are multiple time intervals in the prior period and multiple time intervals in the postperiod. Also imagine that both within the prior period and within the postperiod there is significant association between the lagged and present response, but there is no such association across the prior and postperiods. In this hypothetical situation the DRIM may show significant association between responses in the intervals although the PERR assumption is not violated. However, such a situation is implausible at least in the untreated controls because the separation of the prior and postperiods among the untreated controls occurs only in data analysis, not in real-life. As such, while the DRIM does not directly test this assumption, we maintain that it can evaluate the plausibility of this assumption.

No evidence of assumption violation does not equal evidence of validity of assumptions. In the case study, considering that the point estimates concerned were fairly close to the null value and their confidence intervals excluded strong association, we consider the two assumptions no barrier to the use of PERR. As with any statistical research, careful interpretation of the analytic results is important.

For completeness of illustration we applied the PERR to estimate PCV effect on clinical pneumonia. We caution against a substantive interpretation of this PERR result. This is because there is still uncertainty in not only the evaluation of other assumptions of PERR, such as time-varying confounding, but also how to implement some of the PERR procedures, such as how best to perform matching without losing many persons due to failure to identify a match or more treated than untreated persons. Matching has been an essential procedure in previously published work on PERR for generating index dates for the untreated group. Whether it is always required is a question worthy of investigation. These are areas for future research.

## 5 Conclusion

The PERR is a promising approach for controlling confounding in observational studies and real-world evidence research. The SCCS and DRIM methods can evaluate the plausibility of two of the major assumptions of the PERR and thus facilitate the appropriate use of this approach. However, SCCS and DRIM themselves involve assumptions and should be used with care. Furthermore, there are other assumptions and implementation procedures required by the PERR, for which currently there is a paucity of guidance in the literature.

## Ethics statement

The Gambia Government/MRC Joint Ethics Committee (number 1087) approved the surveillance study.

## CRediT authorship contribution statement

**Yin Bun Cheung:** Writing – review & editing, Writing – original draft, Methodology, Conceptualization. **Xiangmei Ma:** Writing – review & editing, Formal analysis. **Grant Mackenzie:** Writing – review & editing, Investigation, Data curation.

## Declaration of competing interest

There are no competing interests for any author.

## Data Availability

Data collected for the study, including individual, deidentified participant data, and a data dictionary defining each field in the dataset, may be made available to others. Access will be granted following approval of an application for research analysis to the Gambian Government/MRC Joint Ethics Committee.
