# Supplementary material for: Two assumptions of the prior event rate ratio approach for controlling confounding can be evaluated by self-controlled case series and dynamic random intercept modeling
Source: J Clin Epidemiol. 2024 Nov;175:None. doi: 10.1016/j.jclinepi.2024.111511 (PMC11636649; doi:10.1016/j.jclinepi.2024.111511)
Supplement: Supplementary Material [file mmc1.docx]

**Reporting Guideline**

This is a research methodology study. No relevant guideline exists in Equator Network.
