# Supplementary material for: Two assumptions of the prior event rate ratio approach for controlling confounding can be evaluated by self-controlled case series and dynamic random intercept modeling
Source: J Clin Epidemiol. 2024 Nov;175:None. doi: 10.1016/j.jclinepi.2024.111511 (PMC11636649; doi:10.1016/j.jclinepi.2024.111511)
Supplement: Appendix 1 [file mmc2.docx]

**Appendix 1.** Dynamic random intercept model and implementation by Stata *gsem*

1.1. Dynamic random intercept model

Let $y_{i,j}$ be a binary response, $\boldsymbol{z}_{i}$ (with first element equal to 1 for the intercept) be a set of time-constant covariates and $\boldsymbol{x}_{i,j}$ be a set of time-varying covariates for subject $i$ at the *j-*th time-interval. The width of time-interval is constant. The process is observed at times $j=0, 1,\ldots,n_{i}$ for subject $i$, where $j=0$ refers to the first time-interval a study participant is under observation, but the biological / data-generating process may begin prior to $j=0$.^19,21^

Aitkin and Alfo proposed a first-order autoregressive dynamic random intercept model in the form of: ^21^

$\Pr\left( y_{i,j}=1 | y_{i,j-1},\boldsymbol{z}_{i},\boldsymbol{x}_{i,j},\zeta_{i} \right)=h^{-1}\left( \gamma_{1}y_{i,j-1}+\boldsymbol{z}_{i}^{'}\boldsymbol{\beta}_{z}+\boldsymbol{x}_{i,j}^{'}\boldsymbol{\beta}_{x}+\zeta_{i} \right)$ for $j\geq1$

$$\Pr\left( y_{i,0}=1 | \boldsymbol{z}_{i},\boldsymbol{x}_{i,0},\zeta_{i} \right)\approx h^{-1}\left( \boldsymbol{z}_{i}^{'}\boldsymbol{\alpha}_{0z}+\boldsymbol{x}_{i0}^{'}\boldsymbol{\alpha}_{0x}+\lambda_{0}\zeta_{i} \right)$$

where $h(\cdot)$ is the logit link function and$\gamma_{1}$ is the regression coefficient of the first-order lagged response, $y_{i,j-1}$. $\zeta_{i}\sim N(0, \psi)$ is a random-intercept shared by the two equations, with coefficients 1 and $\lambda_{0}$ for $j\geq1$ and $j=0$, respectively. The two equations are to be estimated jointly. The estimation requires different sets of regression coefficients $\left( \boldsymbol{\beta}_{z},\boldsymbol{\beta}_{x} \right)$ and $\left( \boldsymbol{\alpha}_{0z},\boldsymbol{\alpha}_{0x} \right)$ for the covariates in the equations for $j\geq1$ and $j=0$. $\left( \lambda_{0},\boldsymbol{\alpha}_{0z}\boldsymbol{,\alpha}_{0x} \right)$ are nuisance parameters. The parameters $\eta=\left( \gamma_{1}\boldsymbol{,\beta}_{z},\boldsymbol{\beta}_{x},\boldsymbol{\alpha}_{0z},\boldsymbol{\alpha}_{0x},\lambda_{0},\psi\right)$ are to be estimated from the data. This can be achieved by maximum likelihood with adaptive Gauss-Hermite quadrature.

1.2. Stata *gsem* codes for the example study

*** Data in long format. Each person has 6 rows of data in the example.

*** id is unique participant identifier

*** resp is binary outcome

*** age and distance are continuous time-varying covariates

*** edu is categorical and male is binary time-constant covariates

*** Generate lagged response and record number

sort id age

by id: gen ylag=resp[_n-1]

by id: gen record=_n

*** Short name for covariates

global varlist age distance i.edu male

*** Naive model, implemented by melogit

melogit resp ylag $varlist || id: , vce(robust) intp(20)

*** Naive model, implemented by gsem, equivalent to melogit

gsem (resp <- ylag $varlist M1[id]), logit vce(robust) intp(20)

*** Generate variables for dynamic model

gen ylag0 = ylag

replace ylag0 = 0 if record==1

gen nolag0 = cond(record==1,1,0)

gen age_n0 = age*nolag0

gen distance_n0 = distance*nolag0

gen edu_n0 = edu*nolag0

gen male_n0 = male*nolag0

*** Short name for covariates in y0 equation

global varlist0 age_n0 distance_n0 i.edu_n0 male_n0

*** Dynamic model

gsem (resp <- ylag0 $varlist nolag0 $varlist0 M1[id] 1.nolag0#M1[id]@c), ///

logit vce(robust) intp(20)
